# Impact of water deprivation on broilers divergently selected for water efficiency

**DOI:** 10.3389/fphys.2026.1900499

**Published:** 2026-07-31

**Authors:** Rosie H. Whittle, Gregory S. Fraley, Colin G. Scanes, Sara K. Orlowski-Workman, Maricela A. Maqueda, Alexander H. Nelson, Zachary Grider, Shawna L. Weimer

**Affiliations:** 1 Department of Poultry Science, University of Arkansas, Fayetteville, AR, United States; 2 Department of Animal Science, Purdue University, West Lafayette, IN, United States; 3 Department of Biological Sciences, University of Wisconsin Milwaukee, Milwaukee, WI, United States; 4 Department of Electrical Engineering and Computer Science, University of Arkansas, Fayetteville, AR, United States

**Keywords:** behavior, broiler, physiology, water deprivation, water efficiency, welfare

## Abstract

**Introduction:**

Sustainable water use in poultry production is becoming pivotal. Extreme weather events and increased water scarcity may increase the risk of temporary disruptions in freshwater access. To address this challenge, the aim of this study was to determine how broilers selected for low (LWE) and high (HWE) water conversion efficiency responded to water deprivation.

**Methods:**

Straight-run LWE (n=90) and HWE (n=89) broilers were raised in 12 pens (6 pens per line). On D25 and D32, half of the pens were subjected to water deprivation for a minimum of 12 h (deprived), whilst the remaining pens had *ad libitum* access to water (control). Production performance, water intake, body surface temperature, activity, blood chemistry, stress hormones, brain neurotransmitters, and drinking behavior were assessed.

**Results:**

LWE broilers consistently weighed more than HWE (p<0.05), whereas water deprivation did not affect body weight at either time point. From D14 onward, LWE broilers had higher feed and water conversion ratios than HWE (p<0.05). Water intake tended to differ between lines, with LWE broilers having the greater post-deprivation water intake (p=0.06). Water deprivation resulted in lower beak surface temperatures (p<0.001) and changes in blood chemistry consistent with dehydration, with deprived broilers having lower bicarbonate (p=0.01), base excess (p=0.02), and total carbon dioxide levels (p=0.02), and increased sodium levels (p<0.001) than controls. Water deprivation did not affect circulating glucocorticoid concentrations, but it altered central neurochemical activity, primarily through increased serotonin turnover (p<0.05). Drinking behavior did not differ between controls of either line, but HWE-deprived broilers were less likely to remain near drinker nipples immediately after water access was restored than LWE-deprived broilers (p<0.05) on D32.

**Discussion:**

Water deprivation induced physiological signs of dehydration and compensatory drinking behavior, with limited evidence of a stress response, and selection for water efficiency did not result in clear resilience to short-term water deprivation.

## 1 Introduction

Water is an important nutrient in broiler production (National Research Council, 1994; Coon, 2002) and is essential for thermoregulation (Zhou et al., 1999), metabolism (Edge et al., 2025), osmotic-regulation (Lassiter et al., 2025), acid-base homeostasis (Toyomizu et al., 2005), and welfare (Manning et al., 2007). The water demand of a commercial broiler flock is substantial, with drinking water consumption averaging 14,629 liters per 1,000 birds from day 8 to 63 of age (Edge et al., 2025), indicating that even modest improvements in water efficiency could yield substantial water savings. As pressure on freshwater resources increases, the poultry industry may face growing expectations to reduce water use while maintaining productivity. Although poultry meat has a comparatively low water footprint among major animal protein sources, the scale of broiler production amplifies its total water demand. Life cycle assessments that incorporate direct water use, particularly feed production, estimate a total water footprint of 4,325 liters of water per kilogram of chicken meat (Mekonnen and Hoekstra, 2012).

Water use and broiler water efficiency on farms are influenced by both management practices and bird physiology. Water intake can be influenced by genetic line, sex, and age (Hiltz et al., 2025), whereas drinking water use and wastage can be affected by stocking density and drinker design (Feddes et al., 2002). One way to quantify these differences is by assessing the water conversion ratio (WCR), which is the volume of water required for a broiler to gain one unit of body weight. Similar to feed conversion ratio (FCR), WCR is a useful measure of efficiency in poultry production. Improving WCR could reduce drinking water demand and litter moisture, thereby benefiting production, sustainability, and welfare by lowering the risk of contact dermatitis associated with wet litter (Harms et al., 1977). Although reducing litter moisture is an important management objective, intentional restriction of water access may have negative implications for broiler welfare (Weaver et al., 2025). Consequently, understanding the physiological responses to water deprivation is important for forming management decisions and ensuring that efforts to improve litter quality do not inadvertently compromise bird welfare.

Water deprivation represents a biologically relevant stressor for modern broilers because short-term interruptions in water access can occur in commercial production systems despite efforts to provide continuous water availability. Such interruptions may occur during transportation and lairage (Wurtz et al., 2024), as well as through equipment malfunction, management errors, or weather-related events. Because these events can affect large numbers of birds simultaneously, understanding the consequences of short-term water deprivation is important for maintaining bird health and welfare. Water deprivation challenges physiological systems responsible for maintaining fluid and electrolyte homeostasis. Reduced water intake can increase osmolality (Vanderhasselt et al., 2013) and stimulate water-conservation mechanisms, while also altering nutrient intake through reduced feed intake (Ross et al., 1981). These changes may influence acid–base balance (Toyomizu et al., 2005), thermoregulation (Zhou et al., 1999), and activity patterns and motivation to access water (Rault et al., 2016). Although the physiological consequences of prolonged water deprivation (24–48 h) have been reviewed previously (Wurtz et al., 2024), less is known about physiological and behavioral responses to shorter periods of water deprivation that may better resemble some commercial scenarios. Therefore, this study evaluated the effects of a 12-hour water deprivation period, a duration selected to represent a shorter, but biologically meaningful challenge while remaining within institutional ethical guidelines. Understanding responses to short-term water deprivation may help identify welfare risks associated with interruptions in water access and inform strategies to mitigate their impacts.

One potential avenue to mitigate water deprivation stress is genetic selection for water efficiency. Previous research on broilers divergently selected for water efficiency has identified important physiological differences between the lines. Broilers selected for high water efficiency (HWE) exhibit increased metabolic water production, greater water absorption in the gastrointestinal tract, and enhanced renal water reabsorption compared with those selected for low water efficiency (LWE, Greene et al., 2024; Lassiter et al., 2025). Despite substantial differences in water use, early generations of HWE and LWE broilers showed no differences in body weight (Aloui et al., 2024; Greene et al., 2024; Orlowski et al., 2024; Lassiter et al., 2025), suggesting that water-use efficiency can be modified independently of body weight gain. Together, these physiological characteristics suggest that HWE may be better equipped to maintain balance during periods of water deprivation.

Selection for water efficiency has been used to model broiler responses to stressors (Greene et al., 2024, 2025; Orlowski et al., 2024; Santamaria et al., 2025); however, important knowledge gaps remain about the behavioral and physiological responses of these birds to reduced water access. To our knowledge, no studies have examined how water efficiency affects broiler responses to water deprivation. Thus, the aim of our study was to investigate the physiological and behavioral responses of HWE and LWE broilers following a minimum of 12 hours of water deprivation. We hypothesized that the HWE line would exhibit greater resilience to water deprivation than LWE broilers, as measured by water intake, activity, thermal responses, blood chemistry, and neurochemical indicators of stress.

## 2 Materials and methods

### 2.1 Animals and housing

Day-of-hatch broiler chicks from the 6^th^ generation of selection for low (LWE) and high (HWE) water efficiency were obtained from the University of Arkansas Poultry Research Station. The selection process began in 2019 using 24 sire families (one male to three females) originating from a 2015 Modern Random Bred population of broiler chickens (Orlowski et al., 2020). Water conversion ratio was assessed for each family and the six families with the highest and lowest WCR were assigned LWE and HWE, respectively. This was repeated for subsequent generations with WCR used as the single selection trait. A total of 179 straight-run chicks from the LWE (n = 90) and HWE (n = 89) lines were wing banded for identification and randomly allocated to 12 floor pens with fresh pine wood shaving litter (6 pens per line). Pens measured 3.0 × 1.5 m, and 15 chicks were placed in each pen originating from a single genetic line. The initial stocking density was 3.33 birds/m^2^ with the average final stocking density of 1.52 birds/m^2^. Feed was provided *ad libitum* from two hanging feeders, with the starter diet (3050 ME kcal/kg, 23.3% CP) fed from day (D) 0–13 and the grower diet (3125 ME kcal/kg and 21.1% CP) from D14-33. Diets were formulated to meet or exceed industry minimum guidelines. Water was provided through a two-nipple drinker line attached to a low flow water monitoring system that contains four reservoirs attached to a pressure regulator box allowing for adjustable pressure regulation within the pens (Hiltz et al., 2021). Light schedule was set to: D0-2–23 h light: 1h dark (1900 h to 2000 h), D3-6–20 h light: 4 h dark (1900 h to 2300 h), D7-10–18 h light: 6 h dark (1900 h to 0100 h), and D11-33–16 h light: 8 h dark (1900 h to 0300 h). Light intensity was 60 Lux (80 Gallilux) throughout the study.

All procedures were approved by the University of Arkansas Division of Agriculture Institutional Animal Care and Use Committee.

### 2.2 Experimental design

This study utilized a 2 x 2 factorial design, with line (HWE and LWE) and water deprivation treatment (control and deprived) as fixed factors and pen as the experimental unit. On D25 and D32, six pens were subjected to 12 h of water deprivation (deprived); the same pens were deprived at both ages. This resulted in three pens per line × treatment combination. Water access was restricted in the deprived pens by raising the water lines out of bird reach at 0230 h, prior to lights on at 0300 h. On D25, water was returned after 12 h of water deprivation by lowering the water lines in deprived pens at 1430 h. On D32, a subsample of broilers were sampled after 12 h of water deprivation. Water was then returned at 1730 h, after all sampling had been completed, corresponding to 15 h of water withdrawal. The control pens had *ad libitum* water throughout the study.

### 2.3 Data collection and sampling

#### 2.3.1 Production

Individual body weights were recorded on D7, 14, 21, 28, and 33. Feed and water intake during the starter (D0-14) and grower (D14-33) periods were recorded, and mortality-adjusted FCR and WCR were calculated. Water reservoirs were weighed at the start and end of each water deprivation period on D25 and 32, and again 3 h after the water access was restored in deprived pens.

#### 2.3.2 Accelerometry

On D28, accelerometers were placed on the same five focal birds per pen (n = 30 birds/treatment). Triaxial accelerometers (AX3, Axivity Ltd., Newcastle upon Tyne, UK) were secured to harnesses (Vibrant Life, San Francisco, CA, USA) that were adjusted to fit each bird. Broilers were closely monitored, and accelerometers were removed if they did not acclimate to the harness within 24 h. After acclimation, 40 birds with accelerometers remained (control: LWE = 12, HWE = 7; deprived: LWE = 8, HWE = 13). This unequal design was accommodated statistically within a Generalized Additive Mixed Model framework, which is robust to unbalanced designs and estimates effects using all available data (Wood, 2020). The accelerometers were removed on D32 after water deprivation, and data were downloaded using OMGUI Open Movement software (Axivity, Ltd., Newcastle upon Tyne, UK). Acceleration (A_t_) was reported as m/s^2^. To remove the constant of gravitational acceleration (approximately 9.8 m/s^2^), the activity metric was modified by removing a 1g constant from each sample (van Hees et al., 2013):


$$A_{t}=\sqrt{x_{t}^{2}+\;y_{t}^{2}+\;z_{t}^{2}\;}-9.8$$

Acceleration is related to caloric expenditure, and accelerometers measure forces that are proportional to the displacement over a unit of time. The total daily energy expenditure (TDEE) is proportional to the sum of the magnitude of acceleration over time:


$$TDEE\;\propto \;\sum A_{t}$$

Acceleration data were reported as the average magnitude of acceleration on a second-by-second basis, so each data point is the average of approximately 100 measurements per second. This non-physical unit of measurement is used as a proxy for comparing daily energy expenditure. From the processed data, the water deprivation time periods were extracted for each data set. Data intervals were shorter for control than deprived birds due to sampling times. Data were imported into R, combined into a single file, and averaged per minute from the start time, yielding 720 minutes per deprived broiler and 480 minutes per Control broiler. Direct comparisons between treatments were made for the first 480 minutes after treatment. Temporal analysis for deprived broilers included the full 720 minutes to capture the latter hours of water deprivation, where we would expect activity to be most affected by water deprivation.

#### 2.3.3 Thermography

On D25 and D32, thermal images of the head were taken from 5 birds per pen (n = 60) at the end of the 12 h water deprivation period using an infrared camera (Ti480P, Fluke Corporation, Everett, WA, USA), with an emissivity of 0.95 and transmission of 100%. Within each image, the beak region was measured using an ellipse tool to draw an oval in the region of interest (SmartView Classic v4.4), and the maximum beak temperature within the region of interest was recorded. Thermal image analysis was conducted in concordance with Weimer et al. (2020).

#### 2.3.4 Hematology

On D32, control (n=30) and deprived birds (n=30) were sampled at the end of the water deprivation period before water was returned. A small blood sample (~1 mL) was drawn from the brachial vein using a heparinized 3 mL syringe attached to a 2.54 cm 21-gauge needle for blood assays. Immediately after the blood draw, 50 ul of whole blood was analyzed using an iStat CG8+ cartridge, which was inserted into a portable veterinary blood gas analyzer (iStat Alinity, Abbott Laboratories, Chicago, IL, USA) to determine pH, partial pressure of carbon dioxide (PCO_2_), partial pressure of oxygen (PO_2_), sodium (Na^+^), potassium (K^+^), glucose (Glu), hematocrit (Hct), bicarbonate (HCO_3_^-^), total carbon dioxide (TCO_2_), base excess from extra cellular fluids (BE ecf), and hemoglobin (Hb). Then 50 μl of whole blood was analyzed using a hand-held ketone meter (Precision XTRA, Abbott Labs, Columbus, Ohio, USA). Blood was then decanted into plasma separator tubes, centrifuged at 2500 x G for 15 minutes, and plasma was stored at -20 °C until assayed for glucocorticoids. Body weight was recorded, birds were humanely euthanized via manual cervical dislocation by a trained experimenter according to institutional ethical guidelines. The head was subsequently removed as part of the sample collection procedure, confirming death before brain tissue collection. Brains were frozen on dry ice and then stored at -80 °C until microdissection.

#### 2.3.5 Stress hormone ELISAs

Hormone ELISAs (Cayman Chemical, Ann Arbor, MI, USA) have been previously validated for the specificity of each glucocorticoid for use in Galliformes (Abraham et al., 2025). To validate the kits, plasma was charcoal-stripped to remove all steroids, and 3 standard curves were generated for each kit. The first curve was produced using the manufacturer’s ELISA buffer and the respective control steroid. The second standard curve was produced using the steroid for each respective kit, substituting the charcoal stripped plasma in place of the ELISA buffer. The third standard curve was produced again with charcoal-stripped plasma, but with the opposite steroid—cortisol, used with the corticosterone kit, and *vice versa* (Tetel et al., 2022; Abraham et al., 2025). All standard curve ranges were produced following the manufacturer’s recommendation. All remaining steps of the ELISA were completed following the manufacturer’s instructions and plates were read at 405 nm using a plate reader (SynergyLx, Biotek, Winooski, VT, USA).

The Met-enkephalin kit (Enzo Life Sciences, Farmingdale, NY, USA) was validated by spiking plasma samples from chickens of standard genetic lines with identical concentrations matching the kit’s prescribed standard curve. The linearity of these plasma-spiked curves was then compared with the manufacturer’s standard curves.

#### 2.3.6 Brain microdissection

Brains were hemisected into the right and left hemispheres and further microdissected on dry ice. For mass spectrometry, the left or right hemisphere of the brain was processed from each bird, with 50% from the left hemisphere and 50% from the right hemisphere. Hemisected brains were divided into the mesencephalon (MES), diencephalon (DI), lateral telencephalon (LT), and medial telencephalon (MT); right and left hemispheres were randomly used equally among all individuals. Microdissected tissues were weighed and deposited into 2 mL microcentrifuge tubes containing 1.0 mm glass beads, which were stored at -80 °C until used for mass spectrometry.

#### 2.3.7 Brain neurotransmitters quantification

Mass spectrometry was performed following the procedures outlined in mice (Kim et al., 2014) and ducks (Bergman et al., 2024). Briefly, 10 μL of internal standard containing 10 ng/μL of hydrogen heavy isotopes of serotonin (5-HT), 5-Hydroxyindoleacetic acid (5-HIAA), dopamine (DA), 3,4-dihydroxyphenylacetic acid (DOPAC), 3-Methoxytyramine (3-MT), and Homovanillic acid (HVA) were added to the brain samples, and 10 μL per mg of tissue of an acetonitrile and 0.01% ascorbic acid mix was also added. Tissues were then homogenized, vortexed, and centrifuged, and the supernatant was moved to separate 2 mL vials for evaporation in a SpeedVac. Samples were then resuspended, the supernatant was transferred to mass spectrometry tubes, and the tubes were run on an Agilent Triple Quadrupole Mass Spectrometer (QQQ). Neurotransmitter and metabolite concentrations were analyzed using QQQ software.

Neurotransmitter and metabolite concentrations of DA, and 5-HT are reported as concentrations (ng of neurotransmitter/mg of tissue). To determine DA turnover, DA metabolite concentrations (DOPAC, 3-MT, and HVA) were summed and then divided by DA concentration. 5-HT turnover was determined by dividing the 5-HT metabolite (5-HIAA) concentration by the 5-HT concentration (Ahmed and Azmat, 2017; Bergman et al., 2024).

#### 2.3.8 Drinking behavior

Videos were recorded for each pen after water deprivation on D25 and D32 using overhead cameras (Lorex, Markham, Ontario, Canada). Drinking behaviors were observed from videos using scan sampling observations during the 30-minute period immediately after water lines were lowered following deprivation. Control pens were observed concurrently with the deprived pens. To determine the appropriate scan sampling interval, an initial dataset sampled at 5 s was down-sampled to coarser intervals (10–60 s). A 20 s interval was selected based on high agreement with the original data (r≥0.80), resulting in 90 scans per pen and age (2160 total scans). Scan sampling was conducted by three observers, and inter-rater reliability was assessed using intraclass correlation coefficients (ICC) calculated for each pair of observers. ICC values ranged from 0.75 to 0.83, and all comparisons were statistically significant (p<0.001), indicating strong agreement among observers. Broilers were classified as drinking when they were standing with their neck stretched and beak within pecking distance of a nipple on the drinker. Proximity to the drinker nipple was also recorded. Using a ruler, bird body length was measured for each pen, and two zones were drawn around the drinker line corresponding to within one and within two body lengths of a drinker nipple.

#### 2.3.9 Statistics

Data were analyzed in R v4.4.1 and RStudio v2024.04.2. Linear mixed-effects models (LME; “lme4,” (Bates et al., 2015)) were used for all normally distributed continuous variables, with pen as a random factor with three pens per line (LWE, HWE) × treatment (control, deprived) combination. Model assumptions were assessed for normality using simulated residuals from “DHARMa” (Hartig, 2020). BW was analyzed weekly; line was the fixed factor for D7, 14, and 21, and the interaction of line and treatment was the fixed factor for D28 and D33. FCR and WCR were analyzed using ANOVA (“car,” Fox and Weisberg, 2019) within the starter (D0–14) and grower (D14–33) periods, with line as the fixed factor for the starter period and line, treatment, and their interaction for D14–33. Thermal imaging data were analyzed using LME with the interaction of line, treatment, and day. Water intake after water deprivation was analyzed separately for control and deprived treatment groups, using ANOVA with line and day as fixed factors and is reported as gram of water intake per kg of body mass. BW on D28 and D32, BW gain (D28–32), relative bursa weight, all blood gas and chemistry variables, and brain neurotransmitter data were analyzed using LME with line and treatment as fixed factors; time of day was included as a covariate for blood variables. Concentrations of ketones were square-root transformed, while those of corticosterone log−transformed. Estimated means were back-transformed for reporting. Outliers were identified in the brain neurotransmitter data within each line x treatment combination using the 1.5 × interquartile range rule and were removed prior to analysis. Sensitivity analyses were conducted by repeating each statistical model with all observations retained and comparing the results with those obtained after outlier removal. Accelerometer and drinking behavior data were analyzed using Generalized Additive Mixed Models (“mgcv,” (Wood, 2020) to evaluate the effects of line and treatment over time, with temporal variation expressed as effective degrees of freedom (edf), which reflect data variability. Drinking behavior was analyzed using quasibinomial distribution and reported as the predicted probability. Accelerometer data were modelled with the bam() function. Separate smooth functions of time were fitted for each line × deprivation combination. Bird identity was included as a random effect to account for repeated measurements within individuals. Because birds were housed within treatment-specific pens, pen was included as an additional random effect to account for clustering within pens. Residual temporal autocorrelation was assessed using autocorrelation function plots and accommodated using a first-order autoregressive (AR1) correlation structure. Models were fitted using restricted maximum likelihood (fREML). *Post hoc* comparisons were generated using “emmeans” (Lenth and Piaskowski, 2025) and are reported as estimated means ± SE, using Kenward-Roger adjustments for multiple comparisons. Pairwise correlations for individual data were calculated using Pearson’s correlation coefficient and p-values were generated using the “Hmisc” package (Harrell Jr, 2003). To account for multiple testing, p-values were adjusted (q-values) using the Benjamini-Hochberg false discovery rate procedure (Benjamini and Hochberg, 1995). The resulting correlation matrix was visualized with hierarchical clustering (Ward’s method) to group related variables using “ggcorrplot” package (Kassambara, 2016). Correlation matrices were used to assess patterns of system-level coordination across measures, rather than to imply direct mechanistic relationships. Significance was defined as p<0.05, with 0.05<p<0.10 considered a tendency.

## 3 Results

### 3.1 Production

LWE broilers consistently weighed more than HWE broilers (**Table 1**). Water deprivation on D25 and D32 did not affect body weight of deprived compared to controls on D28 or D33. FCR did not differ between lines from D0 to D14, but did from D14 to D33, where LWE broilers had higher FCR than HWE (1.79 ± 0.03 vs. 1.60 ± 0.03, p<0.001). Deprived broilers had higher FCR between D14 and D33 compared to controls (1.75 ± 0.03 vs. 1.64 ± 0.03, p=0.03). There was a trend towards a line x treatment interaction for D14 to D33 FCR (p=0.08), where LWE-deprived broilers had higher FCR than HWE-deprived broilers (1.88 ± 0.04 vs. 1.62 ± 0.04, p=0.006). As expected, LWE had higher WCR than HWE from D0 to D14 (3.17 ± 0.10 vs. 2.53 ± 0.10, p<0.001) and D14 to D33 (4.37 ± 0.22 vs. 2.93 ± 0.22, p=0.002).

**Table 1:** Body weight (BW, g), feed conversion ratio (FCR), and water conversion ratio (WCR) of broilers selected for low (LWE) or high (HWE) water efficiency.

Pre water deprivation	LWE		HWE		Line p-value	Treatment p-value	Line x Treatment p-value
BW D0	45.0 ± 0.43		43.8 ± 0.42		0.06		
BW D7	137 ± 3.65^a^		121 ± 3.69^b^		0.002		
BW D14	369 ± 9.29^a^		324 ± 9.35^b^		<0.001		
BW D21	732 ± 16.6^a^		685 ± 16.8^b^		0.04		
FCR D0-14	1.43 ± 0.03		1.47 ± 0.03		0.39		
WCR D0-14	3.17 ± 0.10^a^		2.53 ± 0.10^b^		<0.001		
Post water deprivation	Control	Deprived	Control	Deprived	Line p-value	Treatment p-value	Line x Treatment p-value
BW D28	1252 ± 30.6	1253 ± 29.3	1179 ± 31.1	1137 ± 30.3	0.002	0.50	0.48
BW D33	1567 ± 54.7	1557 ± 52.1	1496 ± 53.7	1390 ± 51.3	0.02	0.26	0.36
FCR D14-33	1.70 ± 0.04^ab^	1.88 ± 0.04^a^	1.59 ± 0.04^ab^	1.62 ± 0.04^b^	0.001	0.03	0.08
WCR D14-33	4.31 ± 0.32	4.44 ± 0.32	2.95 ± 0.32	2.91 ± 0.32	0.002	0.89	0.80

^ab^ Different superscript letters indicate significant pairwise contrasts.

Birds were either deprived of water for 12 h (deprived) or maintained with continuous water access (control) on days 25 and 32. Pre-deprivation performance is shown through day 21, and post-deprivation performance through day 33.

**Figure 1:**
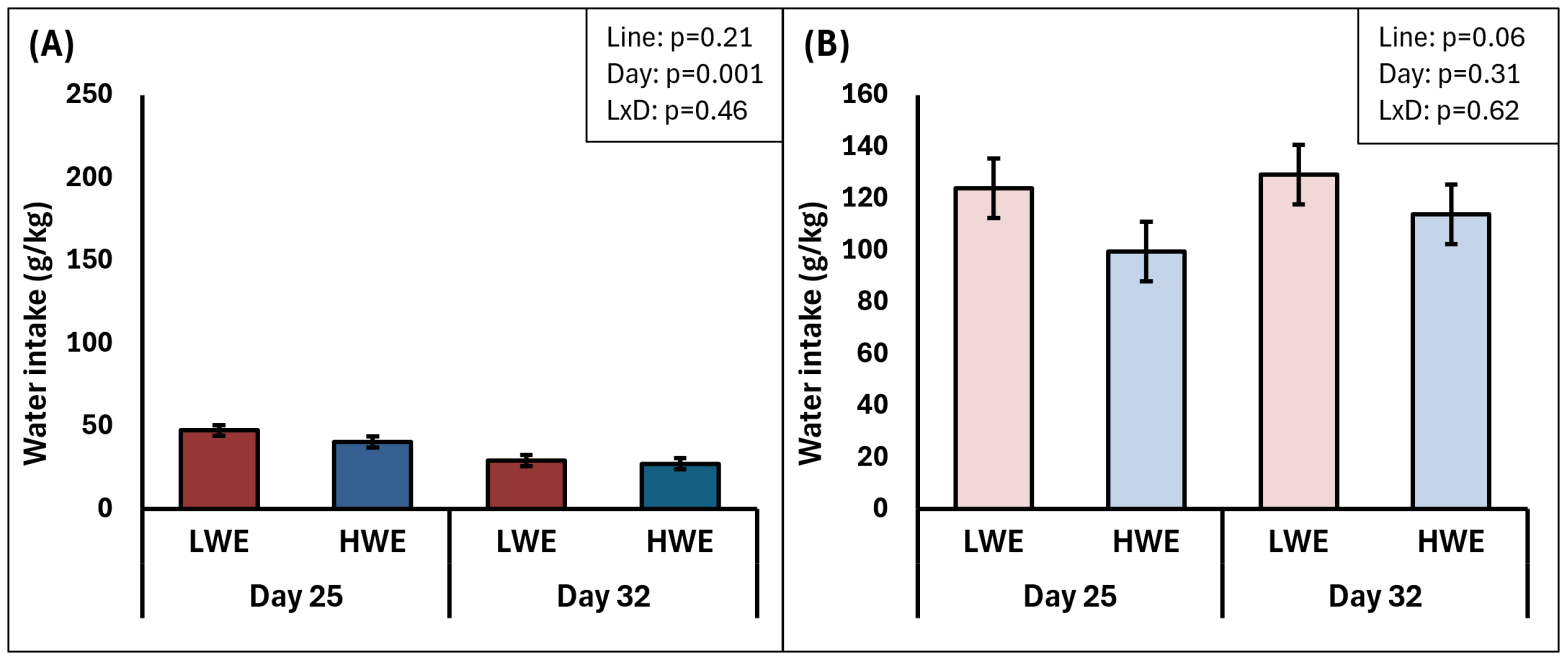
Water intake (g/kg bird) of broilers selected for low (LWE) and high (HWE) water efficiency over a 3 h period after water access was restored. Birds were either **(A)** control with continuous water access or **(B)** water-deprived for 12 h (deprived) prior to water access.

**Figure 1** shows the water intake on D25 and D32 for control (**Figure 1A**) and water deprived (**Figure 1B**) broilers during a 3 h period after water access was returned to deprived pens. Within the control group, when adjusted for mass, there was no difference in water intake between LWE and HWE broilers (38.3 ± 2.31 vs. 33.9 ± 2.31 g/kg). Controls drank more on D25 than D32 (43.9 ± 2.31 vs. 28.2 ± 2.31 g/kg, p=0.001). Within the deprived group, LWE broilers tended to drink more water than HWE (127 ± 6.45 vs. 107 ± 6.45 g/kg, p=0.06), but water intake did not differ between D25 and D32 when adjusted for mass (112 ± 6.45 vs 122 ± 6.45, p=0.31).

### 3.2 Accelerometry

**Figure 2:**
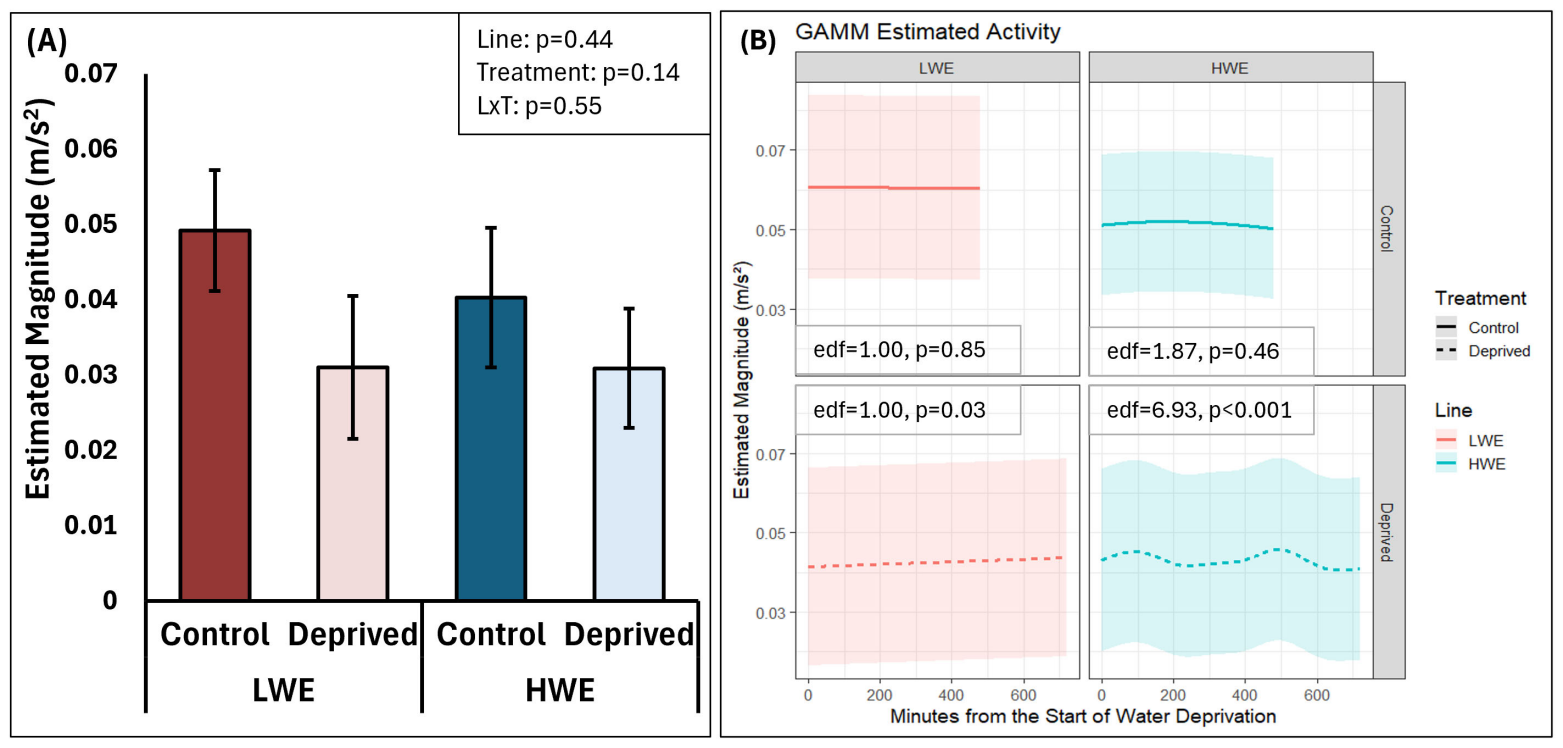
Activity magnitude (m/s^2^) of broilers selected for low (LWE) or high (HWE) water efficiency during water deprivation. **(A)** Mean estimated magnitude over the first 480 min comparing control (continuous water access) and water-deprived (deprived for 12 h) broilers. **(B)** Temporal patterns of activity magnitude (m/s^2^) over time during water deprivation for deprived (12h; 720 min) and control broilers (8h; 480 min) on day 32. Variability is described by the effective degrees of freedom (edf), where edf=1 indicates a linear response and edf>1 indicates increasing nonlinearity. Significant changes in activity magnitude over time are indicated by p<0.05.

**Figure 2** shows the average magnitude of activity during the water deprivation treatment period (**Figure 2A**) and the temporal patterns of activity across time (**Figure 2B**). Deprived broilers were numerically less active than controls (0.031 ± 0.006 vs. 0.045 ± 0.065 m/s^2^, p=0.14). Temporal structure was characterized by using effective degrees of freedom (edf), where edf=1 indicates a perfect linear pattern and higher values indicate more complex temporal changes; a significant p-value suggests a change in activity over time. Despite similar activity levels among groups, temporal activity patterns differed (**Figure 2B**). Activity was generally stable throughout the deprivation period in control birds (LWE-control: edf=1.00, p=0.85; HWE-control: edf=1.87, p=0.46). In contrast, LWE-deprived birds showed a linear increase in activity over the deprivation period (edf=1.00, p=0.03), whereas HWE-deprived broilers had irregular and nonlinear temporal variation, where activity generally declined over time (edf=6.93, p<0.001).

### 3.3 Thermography

**Figure 3:**
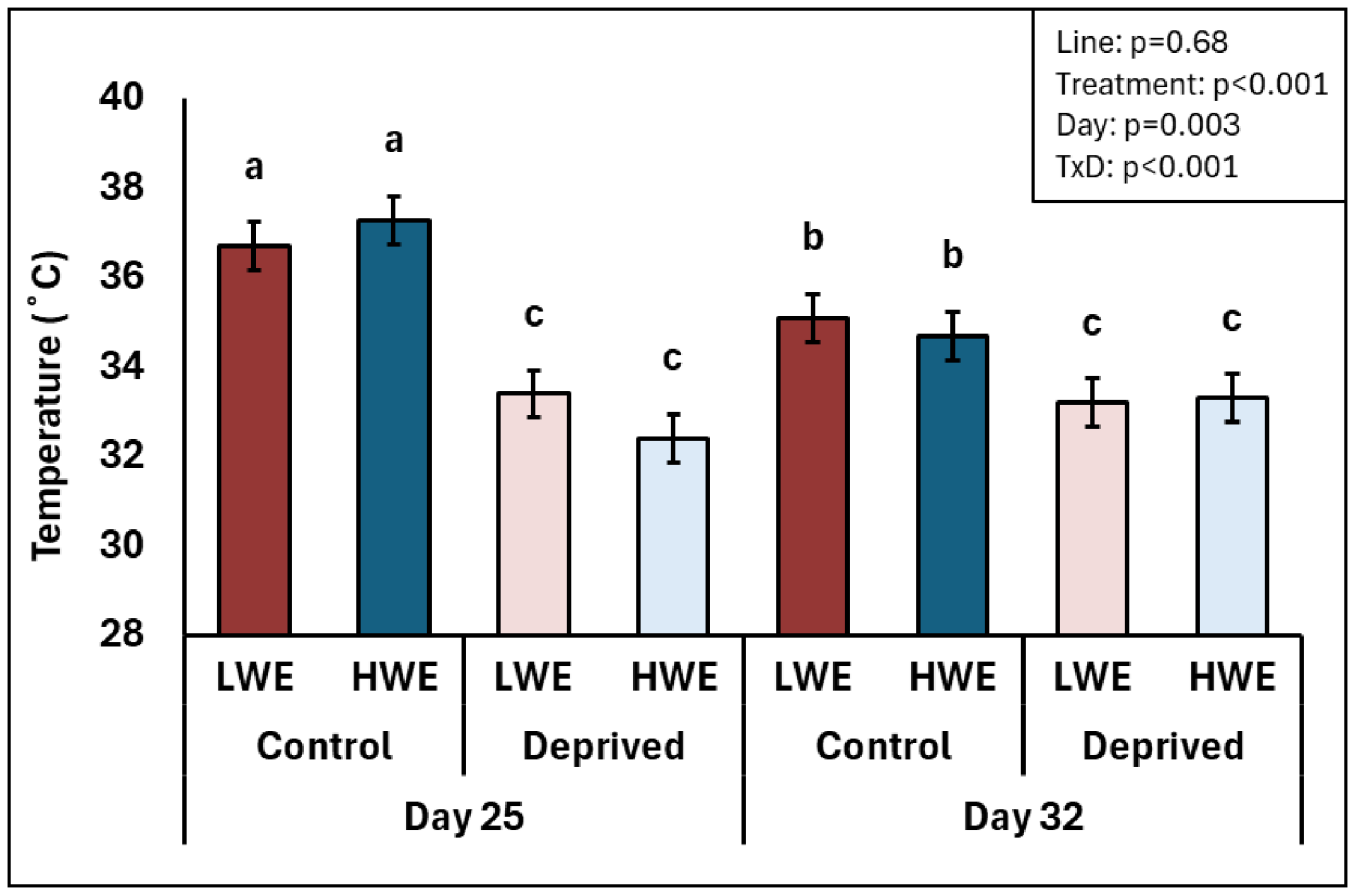
Beak surface temperature (°C) of low (LWE) or high (HWE) water efficiency broilers following 12 h water deprivation (deprived) and controls with continuous water access on days 25 and 32. Different superscript letters (a–c) indicate significant pairwise comparisons for the interaction between day and treatment (p<0.05).

Beak surface temperature was affected by a treatment × day interaction (p<0.001; **Figure 3**). Deprived birds had lower beak temperatures than control birds on D25 (32.9 ± 0.38 vs. 37.0 ± 0.38 °C) and D32 (33.3 ± 0.38 vs. 34.9 ± 0.38 °C). Whereas, control birds had higher beak temperatures on D25 than on D32. Overall, deprived birds had lower beak temperatures than control chickens (p<0.001), and broilers had higher beak temperatures on D25 than D32 (p=0.003). There was no effect of line on beak temperatures (p=0.68).

### 3.4 Hematology

Deprived birds also tended to have decreased PCO_2_ (p=0.06). No effects of deprivation were found for pH and concentrations of potassium (K^+^), glucose, corticosterone, or cortisol. Water-deprived broilers had decreased plasma concentrations of bicarbonate (HCO_3_^-^; 23.5 ± 0.76 vs. 26.9 ± 0.81 mmol/L, p=0.01), base excess (BE; -1.42 ± 0.77 vs. 1.80 ± 0.48 mmol/L, p=0.02), and total carbon dioxide (TCO_2_; 24.7 ± 0.83 vs. 28.2 ± 0.98 mmol/L, p=0.02) compared with control birds.

Water deprivation altered metabolism and electrolyte balance. Deprived broilers had lower circulating ketone concentrations than controls (0.53 ± 0.06 vs. 0.95 ± 0.08 mg/dL, p=0.001) and higher sodium (Na^+^) concentrations (157 ± 1.70 vs. 141 ± 1.75 mmol/L, p<0.001), while plasma Met-enkephalin concentrations tended to be lower in deprived broilers (496 ± 97.8 vs. 785 ± 89.6 pg/mL, p=0.07).

Independent of treatment, line differences were observed in hematological measures. LWE broilers had higher BE (0.94 ± 0.48 vs. -0.55 ± 0.48 mmol/L, p=0.03), hematocrit (24.9 ± 0.43 vs. 23.2 ± 0.43%PCV, p=0.003), and hemoglobin concentrations (8.47 ± 0.15 vs. 7.88 ± 0.15 g/dL, p=0.004) than HWE. No significant line x treatment interactions were detected. Whole blood gas, chemistry, and plasma glucocorticoid concentrations are in **Table 2**.

**Table 2:** Whole blood gas and chemistry, and plasma hormone concentrations of 32-day-old broilers selected for low (LWE) or high (HWE) water efficiency following 12h of water deprivation (*d*eprived) compared with birds with continuous water access (*c*ontrol).

Measure (unit)	LWE Control	LWE Deprived	HWE Control	HWE Deprived	Line p-value	Treatment p-value	Line x Treatment p-value	Sampling hour p-value
pH	7.39 ± 0.02	7.40 ± 0.02	7.37 ± 0.02	7.38 ± 0.02	0.11	0.57	0.81	0.34
PCO_2_ (mmHg)	46.0 ± 2.31	38.2 ± 2.14	44.9 ± 2.27	40.1 ± 2.18	0.80	0.06	0.33	0.40
PO_2_ (mmHg)	45.5 ± 2.64	49.8 ± 2.46	41.9 ± 2.60	48.9 ± 2.50	0.23	0.13	0.36	0.87
HCO_3_^-^ (mmol/L)	27.9 ± 0.95^y^	23.5 ± 0.88^z^	25.8 ± 0.93^y^	23.5 ± 0.90^z^	0.12	**0.01**	0.11	0.66
BE ecf (mmol/L)	3.12 ± 0.96^ay^	-1.25 ± 0.89^az^	0.49 ± 0.95^by^	-1.59 ± 0.91^bz^	**0.03**	**0.02**	0.09	0.84
TCO_2_ (mmol/L)	29.2 ± 1.04^y^	24.6 ± 0.97^z^	27.2 ± 1.03^y^	24.8 ± 0.99^z^	0.22	**0.02**	0.13	0.54
Na^+^ (mmol/L)	140 ± 2.03^z^	158 ± 1.94^y^	141 ± 1.99^z^	155 ± 1.98^y^	0.50	**0.001**	0.13	0.18
K^+^ (mmol/L)	4.83 ± 0.20	5.07 ± 0.19	4.91 ± 0.20	5.35 ± 0.20	0.19	0.25	0.48	0.07
Glucose (mg/dL)	239 ± 6.33	236 ± 5.87	238 ± 6.22	238 ± 5.98	0.83	0.85	0.73	0.17
Hematocrit (%PCV)	24.2 ± 0.89^a^	25.7 ± 0.83^a^	23.3 ± 0.88^b^	23.1 ± 0.84^b^	**0.003**	0.62	0.17	**0.05**
Hemoglobin (g/dL)	8.23 ± 0.30^a^	8.71 ± 0.28^a^	7.92 ± 0.30^b^	7.84 ± 0.29^b^	**0.004**	0.65	0.17	**0.04**
Ketone (mg/dL)	0.92 ± 0.09^y^	0.54 ± 0.07^z^	0.98 ± 0.11^y^	0.52 ± 0.07^z^	0.83	**0.001**	0.50	0.32
Corticosterone (pg/mL)	230 ± 35.6	258 ± 38.2	262 ± 39.0	224 ± 33.7	0.94	0.92	0.23	0.27
Cortisol (pg/mL)	164 ± 26.6	178 ± 25.9	151 ± 27.1	148 ± 26.4	0.23	0.88	0.64	0.51
Met-enkephalin (pg/mL)	778 ± 105	555 ± 113	792 ± 103	438 ± 112	0.52	0.07	0.39	**0.02**

Different superscript letters indicate significant pairwise contrasts for line (a, b) and treatment (y, z) (p<0.05). PCO2, partial pressure of carbon dioxide, PO2, partial pressure of oxygen, HCO3, bicarbonate, BE ecf, base excess in extracellular fluids, TCO2, total carbon dioxide, Na, sodium, K, potassium.

Bolded numbers indicate p<0.05.

### 3.5 Brain neurotransmitters

**Figure 4:**
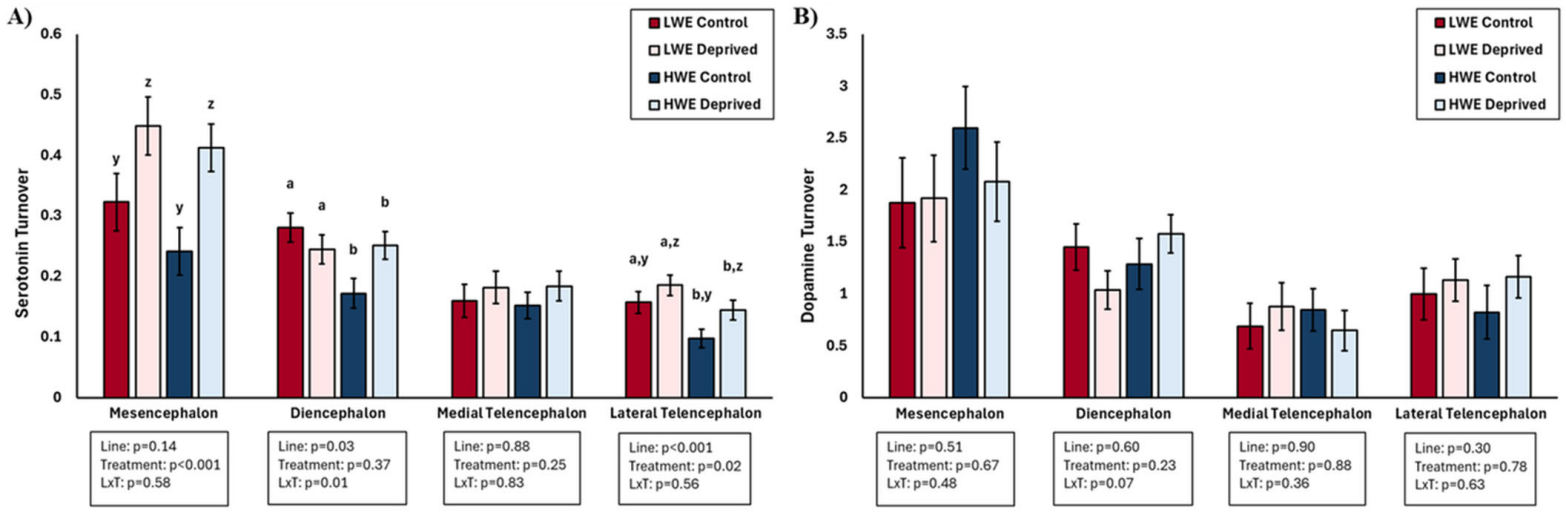
Serotonin **(A)** and dopamine **(B)** turnover in the mesencephalon, diencephalon, medial telencephalon, and lateral telencephalon low (LWE) or high (HWE) water efficiency broilers following 12 h of water deprivation (deprived) and controls with continuous water access on day 32. Superscript letters indicate differences in line (a, b) and treatment (y, z).

Serotonin and dopamine turnovers are directly related to synaptic activity and are presented here and in **Figure 4**. All serotonin, dopamine, and their precursor and metabolite concentrations are presented in **Supplementary Table 1**.

Of the 36 neurotransmitter metrics analyzed, outliers were identified and removed in 22 analyses (**Supplementary Table 2**). Inclusion of outliers altered statistical significance in nine analyses (**Supplementary Table 3**), whereas all remaining analyses yielded the same overall conclusions. Specifically, in the mesencephalon, the effect of line on HVA and 5-HIAA concentrations differed depending on outlier inclusion. In the diencephalon, the effect of line on serotonin and dopamine turnover and treatment on L-Dopa were sensitive to outlier removal. In the medial telencephalon, the effect of treatment on serotonin turnover and on DOPAC and 5-HIAA concentrations differed depending on whether outliers were included. Finally, in the lateral telencephalon the effect of line on HVA was sensitive to outlier removal.

In the mesencephalon, serotonin turnover was greater in deprived broilers than in control broilers (0.43 ± 0.03 vs. 0.28 ± 0.03, p < 0.001), whereas dopamine turnover did not differ among groups. In the diencephalon, LWE broilers had greater serotonin turnover than HWE broilers (0.26 ± 0.02 vs. 0.21 ± 0.02, p = 0.03). A line × treatment interaction was detected for diencephalon serotonin turnover (p = 0.01), with HWE-control broilers having the lowest turnover and LWE-control broilers having the highest turnover; however, Tukey-adjusted pairwise comparisons did not detect significant differences among individual groups. No line, treatment, or line × treatment effects were detected for serotonin or dopamine turnover in the medial telencephalon. In the lateral telencephalon, LWE broilers had greater serotonin turnover than HWE broilers (0.17 ± 0.01 vs. 0.12 ± 0.01, p < 0.001), and deprived broilers had greater serotonin turnover than control broilers (0.17 ± 0.01 vs. 0.13 ± 0.01, p = 0.02). Dopamine turnover did not differ among groups in the lateral telencephalon.

Water deprivation also increased L-Dopa concentrations in the mesencephalon and lateral telencephalon, HVA concentrations in the diencephalon, and 5-HIAA concentrations in the mesencephalon, diencephalon, and lateral telencephalon (**Supplementary Table 1**).

### 3.6 Drinking behavior

**Figure 5:**
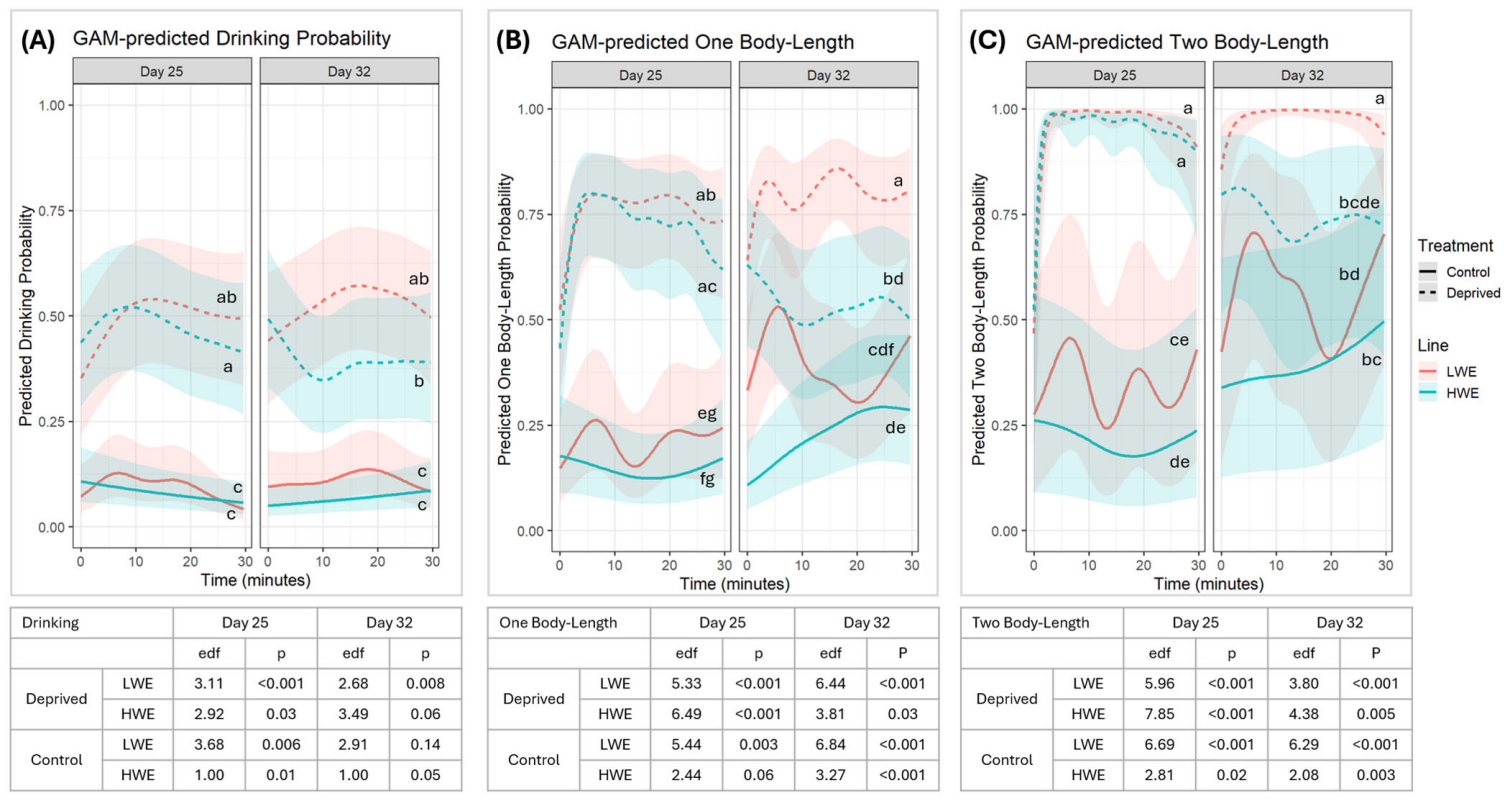
Generalized additive mixed model (GAMM)-predicted probabilities of the drinking behavior of broilers selected for high (HWE) or low (LWE) water efficiency **(A)** drinking, **(B)** being within one body length of the drinker nipple, and **(C)** being within two body lengths of the drinker nipple during the first 30 minutes following water return after a 12 h water deprivation period (deprived) compared to control with continuous water access on days 25 and 32. Birds were housed in three pens per line × treatment combination, with approximately 12 birds per pen on day 25 and seven birds per pen on day 32. Predictions are shown over time, and different letters indicate significant pairwise comparisons from GAMMs for the three-way interaction of line x treatment x day within each behavioral category (p<0.05). Shaded areas indicate the 95% confidence interval. Temporal variation is expressed through the effective degrees of freedom (edf), where edf=1 indicates a linear pattern and higher edf values indicate increased temporal variability. A significant p-value indicates a significant change in probability over time.

Generalized additive mixed models (GAMMs) were used to estimate the predicted probability of broilers drinking (**Figure 5A**), being positioned within one body length of a drinker nipple (**Figure 5B**), and being positioned within two body lengths of a drinker nipple (**Figure 5C**) during the first 30 minutes after water access was returned to the deprived group on D25 and D32.

Across lines and days, deprived broilers were more likely to drink than control chickens (0.49 vs. 0.09, p<0.001). Drinking probability increased rapidly in deprived birds during the first minutes of access, whereas control chickens from both lines maintained consistently low probabilities throughout the observation period (**Figure 5A**). HWE birds were more likely to be drinking on D25 than on D32 (0.22 vs. 0.17, p=0.01). Temporal patterns differed across lines and days. LWE-deprived broilers showed a rapid and sustained increase in drinking probability, whereas HWE-deprived broilers had a pronounced early peak followed by a decline, particularly on D32. Control birds showed minimal changes in drinking behavior over time across lines and days.

Deprived broilers were also more likely to be within one body length of the drinker than in controls (0.73 vs. 0.21, p<0.001). Proximity patterns changed rapidly early in the period. In deprived birds from both lines, proximity to the drinker increased early, then gradually declined, and this effect was strongest on D25. On D32, in HWE−deprived broilers proximity to drinkers was lower than LWE-deprived broilers (0.51 vs. 0.85, p=0.01). Moreover, proximity to drinkers in HWE−deprived broilers was more similar to that in controls (0.51 vs 0.24—0.35, p>0.11), whereas LWE-deprived broilers remained near the drinker for a longer duration (**Figure 5B**). Control birds exhibited more variable proximity patterns across days.

When expanding proximity within two body lengths of the drinker, nearly all deprived broilers were within two body lengths of the drinker (0.98 vs. 0.33, p<0.001) and did so within the first 3 minutes following water return (**Figure 5C**). On D25, this proximity persisted for about 20 minutes. On D32, a higher proportion of LWE−deprived broilers remained near the drinker for a longer period than HWE-deprived (0.99 vs. 0.69, p<0.001), whereas HWE−deprived broilers again displayed proximity patterns similar to control chickens (0.69 vs. 0.38—0.56, p>0.46; **Figure 5C**). Control birds showed lower proximity probabilities overall, with fluctuating, gradual changes over time across lines and days.

### 3.7 Correlations

Correlation matrices for control and deprived broilers are in **Supplementary Figure 1** and were used to assess coordination among physiological, neurochemical and behavioral systems within each line under baseline and dehydration conditions. Under control conditions, both LWE and HWE broilers showed a conserved cluster of acid-base relationships (TCO_2_, HCO_3_^-^, BE, pH and PCO_2_). However, line differences occurred beyond this core cluster. LWE broilers also showed significant correlations between monoaminergic and physiological variables (r=0.81—0.91, q<0.04), whereas HWE broilers had associations linking activity with plasma concentrations of glucocorticoids and ketones (r=-0.77—-0.89, q<0.04).

Following water deprivation, line differences in system coordination became more pronounced. In HWE broilers, plasma concentrations of corticosterone were strongly correlated with acid-base (r=0.54—0.59 and r=-0.56—0.59, q<0.04), electrolyte (r=-0.67, q=0.007) and hematological variables (r=-0.61—-0.75, q<0.01). These structured associations suggest tighter integration between stress signaling and osmotic regulation in HWE broilers. In LWE broilers, significant associations were primarily observed between activity and brain monoamine turnover (r=-0.99, q<0.001).

## 4 Discussion

Our study investigated whether selection for improved water efficiency alters broiler responses to 12 hours of water deprivation; this being an increasingly relevant challenge under conditions of water scarcity. Water deprivation induced physiological changes consistent with dehydration and elicited compensatory drinking. As expected, selection for water efficiency improved water conversion ratio outside of the deprivation periods but did not appear to confer enhanced tolerance to water deprivation. Three pens per line × treatment combination were used in this study, which may have limited the ability to detect subtle deprivation effects. Although overall physiological and performance responses were largely similar among genetic lines, differences in drinking behavior and neurochemical activity indicate that selection for water efficiency may influence how birds respond to water deprivation, rather than preventing dehydration. Our results indicate that selection for water efficiency and resilience to water deprivation are not equivalent traits and highlight that evaluating welfare-relevant responses alongside water use is important.

### 4.1 Physiological indicators of dehydration

Water deprivation altered several blood parameters associated with electrolyte and acid-base balance in broilers. Even under control conditions, both LWE and HWE broilers showed a conserved cluster of acid-base relationships, reflecting core homeostatic regulation. As expected, deprived broilers tended to showed increased circulating sodium concentrations, which is a well-established physiological indicator of dehydration and has previously been reported in broilers restricted water access during transport to slaughter (Vanderhasselt et al., 2013). In rats, serum sodium concentrations plateau within the first 12 hours of water deprivation but the upregulation of the renin-angiotensin system does not peak until after 36 hours (Barney et al., 1983). Future studies in these genetic lines should extend this work by examining additional markers of dehydration and osmoregulatory activation, such as circulating levels of angiotensin II. This lag between responses highlights the potential value of assessing angiotensin-related pathways in broilers, particularly given evidence that hypothalamic expression of angiotensinogen and angiotensin II receptor genes is lower overall in HWE lines (Aloui et al., 2024), while renal expression is upregulated in HWE compared to LWE lines (Lassiter et al., 2025). These differences could be further exacerbated under water deprivation conditions.

Water deprivation also resulted in decreased plasma concentrations of bicarbonate, base excess, and total carbon dioxide. These changes are consistent with altered acid-base buffering capacity and suggest a disturbance in acid-base regulation. Altered acid-base balance has been studied in relation to heat stress and linked to dehydration (Zhou et al., 1999; Toyomizu et al., 2005). To our knowledge, ours is the first study to specifically characterize these acid-base changes in broilers subjected to water deprivation alone, independent of thermal stressors. Despite reductions in bicarbonate, base excess, and total carbon dioxide, we did not detect a significant effect water deprivation on blood pH. One possible explanation is that there is a concurrent decrease in PCO_2_, which would tend to raise blood pH through respiratory compensation (Calder and Schmidt-Nielsen, 1966). Reduced PCO_2_ can result from increased respiratory rate or inefficient respiration, both of which promote carbon dioxide loss from the blood (Calder and Schmidt-Nielsen, 1966). Together, these findings suggest that water deprivation induced changes consistent with altered acid-base buffering and possible compensatory regulation, which may have contributed to maintenance of blood pH homeostasis.

### 4.2 Stress

Water deprivation did not affect circulating corticosterone, cortisol, or glucose concentrations. The absence of measurable circulating glucocorticoid response suggests that 12 h of water deprivation did not elicit a classical acute stress response and that glucose homeostasis was maintained under this challenge. However, differences in beak temperature, serotonin turnover, activity and compensatory drinking behavior indicate that broilers nonetheless exhibited physiological and behavioral adjustments in response to water deprivation. Additionally, correlations between serotonin and dopamine turnover and acid-base, electrolyte and hematological measures suggest coordination within the broader endocrine-physiological response to dehydration. Similar patterns have been reported in wild birds (house sparrows), where water deprivation alone did not elevate corticosterone (Beattie et al., 2022). Previous studies indicate that stronger endocrine responses may require the combined effects of water deprivation with feed withdrawal to activate the hypothalamic-pituitary-adrenal axis, in broilers and Japanese quail (Scott et al., 1983).

Circulating concentrations of ketones were lower in water deprived compared to control broilers. Ketones have recently been evaluated as an indicator of oxidative stress in wild birds (Viblanc et al., 2018; Beattie et al., 2022, 2023). Decreases in ketones have been observed in king penguins in response to acute handling stress (Viblanc et al., 2018). Reduced concentrations of ketone may reflect suppressed hepatic fat oxidation due to reduced feed intake or a broader metabolic downregulation during dehydration (Sherlock et al., 2012). Because feed intake was not measured specifically during the deprivation period, we cannot distinguish between direct effects of water deprivation and indirect effects resulting from reduced feed consumption. Therefore, reduced circulating ketones may reflect the combination of metabolic changes due to dehydration and reduced feed intake.

Plasma Met-enkephalin concentrations tended to be lower in deprived broilers. Although concentrations of Met-enkephalin have been shown to increase following acute restraint stress in laying hens (Scanes et al., 2024), its response to more prolonged or chronic stressors is less well understood. Previous work in rodents showed that administering Met-enkephalin prior to a one-hour immobilization attenuated stress-induced noradrenaline release (Tanaka et al., 1989), suggesting that higher circulating Met-enkephalin in control birds reflects greater physiological robustness rather than stress per se.

Water deprivation was associated with changes in peripheral temperature and brain neurotransmitter activity. Broilers deprived of water had lower beak temperatures, consistent with stress-induced peripheral vasoconstriction. Stress-induced hypothermia is part of the fight-or-flight response, where blood is diverted to vital organs, resulting in decreased temperature in the extremities, such as the comb (Edgar et al., 2013) and beak (Tabh et al., 2021). The absence of difference in the eye likely reflects lower temperature variability compared with the beak, making changes less detectable (Tabh et al., 2021). Future work could explore the magnitude of temperature changes relative to basal measures taken within the home pen and following capture to assess whether handling stress compounds the physiological effects of water deprivation.

At the neurochemical level, water deprivation increased serotonin turnover but not dopamine turnover in several brain regions. Serotonin is involved in stress responsiveness, homeostasis and coping systems, and an increase in serotonin turnover may reflect a neurochemical response to water deprivation that has been previously related to a relatively reduced affective state (Blanchard et al., 1991; van Hierden et al., 2004; Buitenhuis et al., 2006; Narvaes and Almeida, 2014; Ahmed and Azmat, 2017; Bergman et al., 2025; Oluwagbenga et al., 2025). The lack of change in dopamine turnover suggests that motivational or reward-related pathways may remain relatively stable under short-term water deprivation, as increased dopamine turnover has been linked to reward pathways and impulse control in feather pecking chickens (van Hierden et al., 2004). Several neurotransmitter measures were sensitive to the outlier-removal procedure (**Supplementary Table 2**), indicating that interpretation of these findings should be made with caution.

### 4.3 Activity during water deprivation

Water deprivation induced marked changes in the temporality of activity, with clear line-specific patterns. Broilers subjected to water deprivation were numerically less active than their control counterparts, however due to large variation within groups this failed to achieve significance. Although the line × treatment interaction for FCR was not statistically significant, the tendency for the highest FCR in LWE-deprived broilers may support the behavioral evidence that LWE birds were more affected by water deprivation. LWE-deprived broilers tended to consume more water after access was restored, remained closer to drinkers for longer on D32, and showed increasing activity during the deprivation period. These patterns suggest that water deprivation may have imposed greater behavioral or energetic costs in LWE broilers, whereas HWE broilers appeared to show a more conservative behavioral response. However, the FCR interaction was only a tendency, and this interpretation is not conclusive. Reduction in activity is also consistent with energy−conserving behavioral adjustments previously reported in water−deprived laying hens (Rault et al., 2016). Notably, activity in LWE broilers increased in an linear pattern over time, whereas HWE broilers showed a general decline in activity. Thus, while HWE broilers responded to water deprivation in the expected manner, by reducing activity, LWE broilers did not exhibit this typical compensatory behavior.

The increased activity observed in LWE broilers may indicate heightened motivation to seek water (Leib et al., 2016) or elevated agitation under deprivation. Evidence from broilers under water restriction associated up to 40% water restriction with redirected frustration behavior, such as increased toe pecking toward conspecifics (Viola et al., 2009). Thus, differences in activity trajectories reflect divergent behavioral coping strategies rather than differences in overall activity levels and lend further support to the possibility that water deprivation can elevate agitation or restlessness in broilers.

### 4.4 Post deprivation drinking behavior and water intake

Post−deprivation water intake was not affected by age when normalized for body weight, whereas control broilers drank less water per body mass on D32 compared to D25. While sex was not determined in this study, an attempt to account for the potential sex-specific responses to water deprivation was made by normalizing for body weight. Sex can influence growth performance (Lassiter et al., 2025), water intake (Hiltz et al., 2025), and gene expression relating to water efficiency (Lassiter et al., 2025). Future research should evaluate whether male and female broilers differ in their physiological responses to water deprivation.

LWE broilers tended to consume more water than HWE broilers, despite no differences in the overall probability of drinking behavior, indicating that behavioral measures alone may not accurately reflect water intake. Differences in proximity to the water source revealed that LWE broilers were more likely to be within one or two body lengths of the drinker on D32. This may reflect greater motivation to remain close to water sources. As previously reported, increased drinking duration and proximity to drinkers following water deprivation in both broilers (Boone et al., 2009) and laying hens (Rault et al., 2016). The longer proximity response in deprived LWE broilers may indicate greater motivation to remain near the water source or greater competition for drinker access after water return. In contrast, deprived HWE broilers showed a pronounced early drinking response followed by a decline in proximity, suggesting a shorter-lived or more rapidly resolved post-deprivation drinking response. These behavioural differences align with the broader correlation patterns observed, whereby HWE broilers exhibited a more coordinated, multisystem response to dehydration, while LWE broilers showed a more fragmented pattern of regulation. A further consideration is that the same pens were subjected to water deprivation on D25 and D32. Thus, responses during the second deprivation age may have been influenced by prior exposure through habituation, learning, or sensitization. This phenomenon may have been partially explained if activity data had been recorded at both ages. More broadly, increased proximity to drinkers may indicate increased social facilitation or resource competition. However, previous work in laying hens has shown that behavioral synchrony can decrease as resources become limited (Meunier-Salaün and Faure, 1984), indicating that proximity does not necessarily reflect coordinated group behavior.

## 5 Conclusion

Overall, 12 hours of water deprivation induced measurable physiological, neurochemical, and behavioral changes in broilers consistent with dehydration and compensatory drinking motivation. These responses were largely consistent between broilers selected for high and low water efficiency, and selection for water efficiency did not confer resilience to short-term water deprivation. However, differences in activity patterns, drinking behavior, neurochemical regulation, and exploratory correlation structure suggest that water efficiency may influence the coordination and integration of physiological and behavioral responses to water deprivation, rather than their overall magnitudes. These results indicate that even relatively short periods of water deprivation can elicit detectable responses in broilers, although birds may tolerate short-term water restriction without activating a measurable glucocorticoid response. Future work is needed to determine whether longer deprivation periods, repeated disruptions, or concurrent environmental stressors provoke more pronounced differences. Understanding the relationship between water efficiency and dehydration tolerance is important for breeding strategies that support both resource efficiency and broiler robustness.

## Data Availability

The raw data supporting the conclusions of this article will be made available by the authors, without undue reservation.

## Supplementary material

Supplementary Figure 1 Correlation matrix based on individual-level measures from high water efficient (upper triangle) and low water efficient (lower triangle) **(A)** control broilers with continuous water access and **(B)** broilers after 12 hours of water deprivation on day 32. N = 15 per line × treatment combination for all comparisons except brain monoamines (N = 6 per line × treatment). Color indicates strength and direction of the correlation, with r=1 in red and r=-1 in blue. P-values were adjusted for multiple testing using the Benjamini-Hochberg false discovery rate, and only significant correlations (q < 0.05 are shown). The measures are as follows: cortisol, met-enkephalin (MetEnk), corticosterone (Cort), ketone, hemoglobin (Hb*), hematocrit (Hct), glucose (Glu), potassium (K^+^), sodium (Na^+^), total carbon dioxide (TCO_2_), base excess (BE_ecf), bicarbonate (HCO_3_^-^), partial pressure of oxygen (PO_2_), partial pressure of carbon dioxide (PCO_2_), pH, beak temperature (Bmax), eye temperature (Emax), mean activity from accelerometers during the water deprivation period, dopamine (LT_DT) and serotonin turnover (LT_ST) in the lateral telencephalon, dopamine (DI_DT) and serotonin turnover (DI_ST) in the diencephalon, dopamine (MES_DT) and serotonin turnover (MES_ST) in the mesencephalon.
