# Supplementary material for: Impact of water deprivation on broilers divergently selected for water efficiency
Source: Front Physiol. 2026 Jul 31;17:1900499. doi: 10.3389/fphys.2026.1900499 (PMC13472827; doi:10.3389/fphys.2026.1900499)
Supplement: Supplementary file 1 [file Table1.docx]

Supplemental Table 1: Dopamine, serotonin, their precursor and metabolite concentrations, and turnover ratios in the mesencephalon, diencephalon, medial telencephalon, and lateral telencephalon of 32-day-old broilers selected for low (LWE) or high (HWE) water efficiency following 12h of water deprivation (deprived) compared with birds maintained with continuous water access (controls). N = 6 per line × treatment combination. Values reported as estimated marginal means ± SE. Differing superscript letters represent significant pairwise comparisons (p < 0.05) by line (a, b) or treatment (x, y, z)

|  | **LWE**  **Control** | **LWE**  **Deprived** | **HWE**  **Control** | **HWE**  **Deprived** | **Line** | **Treatment** | **Line x Treatment** |
| --- | --- | --- | --- | --- | --- | --- | --- |
| **Mesencephalon** |  |  |  |  | p-value | p-value | p-value |
| Dopamine turnover^1^ | 1.88±0.43 | 2.60±0.40 | 1.92±0.42 | 2.08±0.38 | 0.51 | 0.27 | 0.48 |
| Dopamine (ng/mg) | 0.074±0.01 | 0.060±0.01 | 0.066±0.01 | 0.066±0.01 | 0.95 | 0.55 | 0.55 |
| L-Dopa (ng/mg)^2^ | 0.18±0.12^z^ | 0.52±0.11^y^ | 0.32±0.13^z^ | 0.62±0.11^y^ | 0.31 | **0.005** | 0.86 |
| DOPAC (ng/mg)^3^ | 0.029±0.006 | 0.028±0.005 | 0.028±0.005 | 0.026±0.005 | 0.75 | 0.73 | 0.98 |
| HVA (ng/mg)^4^ | 0.059±0.013 | 0.072±0.011 | 0.051±0.011 | 0.071±0.010 | 0.74 | 0.15 | 0.78 |
| 3-MT (ng/mg)^5^ | 0.041±0.015 | 0.051±0.015 | 0.032±0.017 | 0.036±0.014 | 0.41 | 0.66 | 0.84 |
| Serotonin turnover^6^ | 0.32±0.05^z^ | 0.45±0.05^y^ | 0.24±0.04^z^ | 0.41±0.04^y^ | 0.14 | **<0.001** | 0.58 |
| Serotonin (ng/mg) | 1.67±0.26 | 1.71±0.23 | 1.31±0.23 | 1.38±0.22 | 0.13 | 0.80 | 0.93 |
| 5-HIAA (ng/mg)^7^ | 0.53±0.08^z^ | 0.56±0.09^y^ | 0.31±0.07^z^ | 0.56±0.07^y^ | 0.08 | **0.04** | 0.13 |
| **Diencephalon** |  |  |  |  |  |  |  |
| Dopamine turnover | 1.45±0.22 | 1.29±0.24 | 1.04±0.18 | 1.58±0.18 | 0.60 | 0.23 | 0.07 |
| Dopamine (ng/mg) | 0.16±0.04 | 0.16±0.05 | 0.18±0.04 | 0.19±0.04 | 0.94 | 0.51 | 0.98 |
| L-Dopa (ng/mg) | 0.18±0.11 | 0.44±0.11 | 0.35±0.12 | 0.44±0.11 | 0.49 | 0.10 | 0.44 |
| DOPAC (ng/mg) | 0.044±0.011 | 0.047±0.011 | 0.043±0.011 | 0.062±0.011 | 0.48 | 0.33 | 0.46 |
| HVA (ng/mg) | 0.12±0.03^z^ | 0.17±0.03^y^ | 0.11±0.03^z^ | 0.17±0.03^y^ | 0.75 | **0.05** | 0.77 |
| 3-MT (ng/mg) | 0.077±0.02 | 0.091±0.02 | 0.068±0.02 | 0.097±0.02 | 0.95 | 0.25 | 0.66 |
| Serotonin turnover | 0.28±0.024^a^ | 0.25±0.024^a^ | 0.17±0.025^b^ | 0.25±0.023^a^ | **0.03** | 0.37 | **0.01**^8^ |
| Serotonin (ng/mg) | 1.25±0.23 | 1.58±0.21 | 1.43±0.20 | 1.46±0.20 | 0.86 | 0.36 | 0.44 |
| 5-HIAA (ng/mg) | 0.28±0.05^z^ | 0.39±0.05^y^ | 0.25±0.05^z^ | 0.35±0.05^y^ | 0.46 | **0.03** | 0.88 |
| **Medial Telencephalon** |  |  |  |  |  |  |  |
| Dopamine turnover | 0.68±0.22 | 0.85±0.21 | 0.88±0.23 | 0.65±0.20 | 0.90 | 0.88 | 0.36 |
| Dopamine (ng/mg) | 1.15±0.45 | 1.18±0.41 | 0.46±0.41 | 1.31±0.39 | 0.51 | 0.28 | 0.31 |
| L-Dopa (ng/mg) | 0.40±0.14 | 0.62±0.13 | 0.39±0.13 | 0.54±0.12 | 0.68 | 0.13 | 0.76 |
| DOPAC (ng/mg) | 0.09±0.04 | 0.12±0.03 | 0.05±0.04 | 0.12±0.04 | 0.59 | 0.20 | 0.56 |
| HVA (ng/mg) | 0.22±0.06 | 0.23±0.05 | 0.10±0.05 | 0.17±0.05 | 0.08 | 0.40 | 0.52 |
| 3-MT (ng/mg) | 0.26±0.08 | 0.37±0.07 | 0.22±0.07 | 0.30±0.07 | 0.41 | 0.16 | 0.77 |
| Serotonin turnover | 0.16±0.03 | 0.18±0.03 | 0.15±0.02 | 0.18±0.02 | 0.88 | 0.25 | 0.83 |
| Serotonin (ng/mg) | 1.97±0.29 | 2.19±0.27 | 1.72±0.30 | 1.71±0.26 | 0.18 | 0.71 | 0.68 |
| 5-HIAA (ng/mg) | 0.25±0.08 | 0.41±0.08 | 0.27±0.08 | 0.37±0.07 | 0.92 | 0.08 | 0.73 |
| **Lateral Telencephalon** |  |  |  |  |  |  |  |
| Dopamine turnover | 1.00±0.25 | 0.83±0.26 | 1.13±0.20 | 1.17±0.20 | 0.30 | 0.78 | 0.63 |
| Dopamine (ng/mg) | 0.34±0.13 | 0.48±0.11 | 0.34±0.11 | 0.32±0.12 | 0.48 | 0.57 | 0.46 |
| L-Dopa (ng/mg) | 0.23±0.07^bz^ | 0.44±0.06^by^ | 0.30±0.06^az^ | 0.62±0.07^ay^ | **0.03** | **<0.001** | 0.34 |
| DOPAC (ng/mg) | 0.035±0.011 | 0.051±0.010 | 0.040±0.009 | 0.050±0.010 | 0.84 | 0.17 | 0.77 |
| HVA (ng/mg) | 0.12±0.02 | 0.14±0.02 | 0.08±0.02 | 0.11±0.02 | 0.06 | 0.15 | 0.71 |
| 3-MT (ng/mg) | 0.22±0.06 | 0.23±0.06 | 0.17±0.05 | 0.21±0.06 | 0.52 | 0.76 | 0.79 |
| Serotonin turnover | 0.16±0.02^az^ | 0.19±0.02^ay^ | 0.10±0.01^bz^ | 0.14±0.02^by^ | **0.001** | **0.02** | 0.56 |
| Serotonin (ng/mg) | 1.70±0.21 | 1.83±0.19 | 1.59±0.18 | 1.69±0.18 | 0.47 | 0.52 | 0.94 |
| 5-HIAA (ng/mg) | 0.27±0.05^az^ | 0.35±0.04^ay^ | 0.15±0.04^bz^ | 0.28±0.04^by^ | **0.02** | **0.01** | 0.52 |

^1^ Dopamine turnover calculated as: (DOPAC + HVA + 3-MT) / Dopamine, ^2^ L-Dopa = L-3,4-dihydroxyphenylalanine, ^3^ DOPAC = 3,4-Dihydroxyphenylacetic acid, ^4^ HVA = Homovanillic acid, ^5^ 3-MT = 3-Methoxytyramine, ^6^ Serotonin turnover calculated as: 5-HIAA / Serotonin, ^7^ 5-HIAA = 5-Hydroxyindoleacetic acid ^8^ No significant pairwise comparisons after Kenward-Roger correction for multiple testing.

Supplemental Table 2: Number of observations identified as outliers and removed using the 1.5 × interquartile range (IQR) rule for each neurotransmitter metric within line × treatment combinations. The final column indicates whether inclusion of outliers altered the statistical inference in sensitivity analyses.

|  | **LWE**  **Control** | **LWE**  **Deprived** | **HWE**  **Control** | **HWE**  **Deprived** | **Total** | **Inference change** |
| --- | --- | --- | --- | --- | --- | --- |
| **Mesencephalon** |  |  |  |  |  |  |
| Serotonin Turnover | 0 | 1 | 0 | 0 | 1 | No |
| Dopamine | 1 | 0 | 0 | 0 | 1 | No |
| HVA^1^ | 3 | 0 | 0 | 1 | 4 | Yes |
| 5-HIAA ^2^ | 0 | 2 | 0 | 0 | 2 | Yes |
| **Diencephalon** |  |  |  |  |  |  |
| Dopamine Turnover | 0 | 2 | 0 | 0 | 2 | Yes |
| Serotonin Turnover | 0 | 2 | 0 | 1 | 3 | Yes |
| Dopamine | 0 | 0 | 2 | 1 | 3 | No |
| DOPAC ^3^ | 0 | 1 | 1 | 1 | 3 | No |
| L-Dopa ^4^ | 0 | 0 | 0 | 1 | 1 | Yes |
| 3-MT ^5^ | 0 | 0 | 1 | 0 | 1 | No |
| 5-HIAA | 1 | 2 | 0 | 1 | 4 | No |
| **Medial Telencephalon** |  |  |  |  |  |  |
| Dopamine Turnover | 1 | 0 | 1 | 0 | 2 | No |
| Serotonin Turnover | 0 | 1 | 0 | 1 | 2 | Yes |
| DOPAC | 0 | 0 | 0 | 1 | 1 | Yes |
| 5-HIAA | 1 | 1 | 0 | 0 | 2 | Yes |
| **Lateral Telencephalon** |  |  |  |  |  |  |
| Dopamine Turnover | 0 | 2 | 0 | 0 | 2 | No |
| Serotonin Turnover | 0 | 1 | 0 | 1 | 2 | No |
| Dopamine | 1 | 0 | 0 | 1 | 2 | No |
| DOPAC | 1 | 0 | 0 | 1 | 2 | No |
| L-Dopa | 0 | 0 | 0 | 1 | 1 | No |
| HVA | 1 | 0 | 0 | 0 | 1 | Yes |
| 3-MT | 0 | 0 | 0 | 1 | 1 | No |

^1^ HVA = Homovanillic acid, ^2^ 5-HIAA = 5-Hydroxyindoleacetic acid, ^3^ DOPAC = 3,4-Dihydroxyphenylacetic acid, ^4^ L-Dopa = L-3,4-dihydroxyphenylalanine, ^5^ 3-MT = 3-Methoxytyramine

Supplemental Table 3: Results of sensitivity analyses comparing statistical models performed with outliers removed and with all observations retained. Only neurotransmitter metrics for which statistical inference differed between analyses are presented where NS = not significant, T = trend towards significance, and S = significant.

| **Region** | **Measure** | **Effect** | **Outliers Removed** | **All Observations** | **Inference Change** |
| --- | --- | --- | --- | --- | --- |
|  |  |  | p-value | p-value |  |
| Mesencephalon | HVA^1^ | Line | 0.74 | 0.02 | NS 🡪 S |
|  | 5-HIAA^2^ | Line | 0.08 | 0.18 | T 🡪 NS |
|  | 5-HIAA | Treatment | 0.04 | 0.08 | S 🡪 T |
| Diencephalon | Dopamine Turnover | Line * Treatment | 0.07 | 0.98 | T 🡪 NS |
|  | Serotonin Turnover | Line | 0.03 | 0.29 | S 🡪 NS |
|  | Serotonin Turnover | Line * Treatment | 0.01 | 0.97 | S 🡪 NS |
|  | L-Dopa^3^ | Treatment | 0.10 | 0.03 | T 🡪 S |
| Medial Telencephalon | Serotonin Turnover | Treatment | 0.25 | 0.06 | NS 🡪 T |
|  | DOPAC^4^ | Treatment | 0.20 | 0.04 | NS 🡪 S |
|  | 5-HIAA | Treatment | 0.08 | 0.22 | T 🡪NS |
| Lateral Telencephalon | HVA | Line | 0.06 | 0.04 | T 🡪 S |

^1^ HVA = Homovanillic acid, ^2^ 5-HIAA = 5-Hydroxyindoleacetic acid, ^3^ L-Dopa = L-3,4-dihydroxyphenylalanine, ^4^ DOPAC = 3,4-Dihydroxyphenylacetic acid
